# The hidden danger behind a normal endoscopy: successful endoscopic full-thickness resection guided by endoscopic ultrasound

**DOI:** 10.1055/a-2638-5906

**Published:** 2025-07-14

**Authors:** Rui Zhong, Yufang Wang, Kui Zhao

**Affiliations:** 1 34753 Department of Gastroenterology and Hepatology, West China Hospital of Sichuan University, Chengdu, China; 2 Department of Gastroenterology, Clinical Medical College and the First Affiliated Hospital of Chengdu Medical College, Chengdu, China

**Fig. 1:**
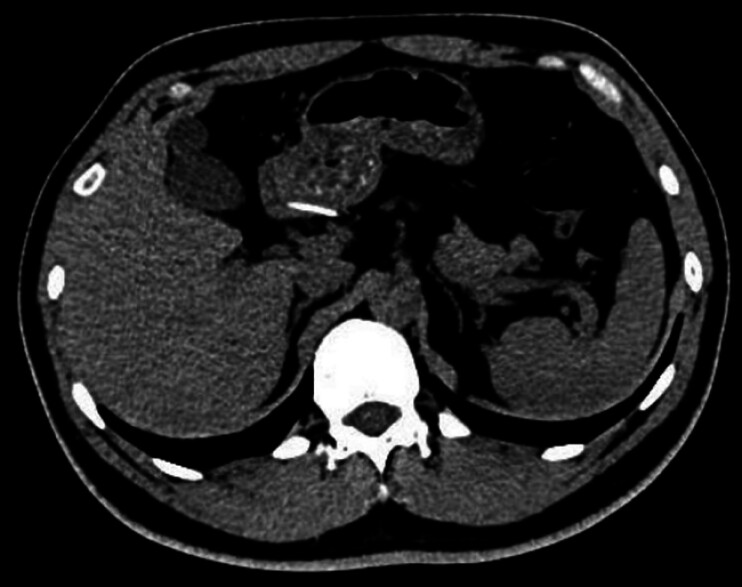
Abdominal computed tomogram showing a linear hyperdense foreign body adjacent to the lesser curvature of the gastric antrum.

**Fig. 2:**
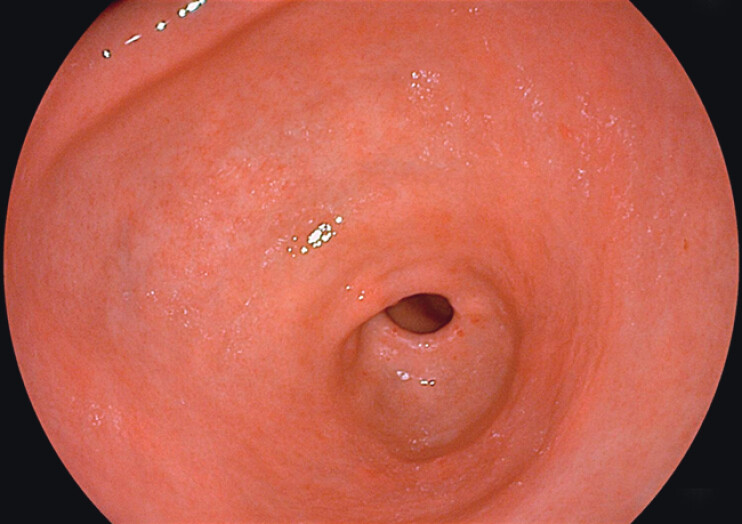
Initial upper endoscopic image showing no visible mucosal abnormalities.

**Fig. 3:**
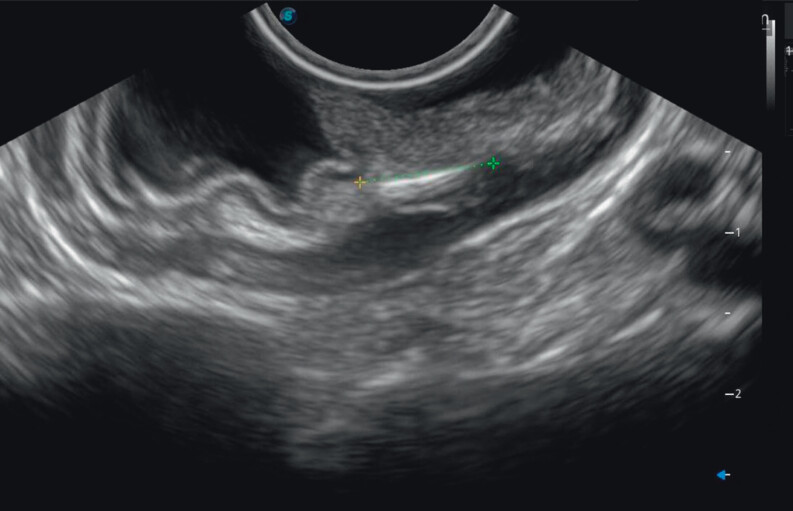
Endoscopic ultrasonogram identifying the location and depth of the fish bone within the muscularis propria.

**Fig. 4:**
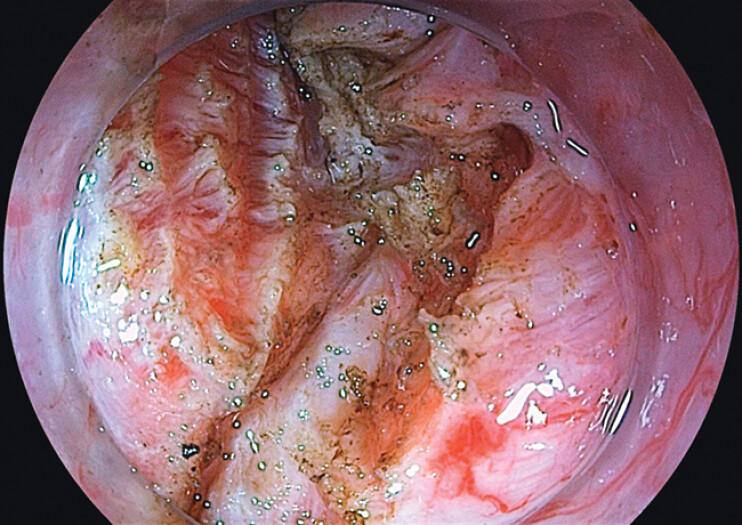
Chronic inflammation and fibrosis suggesting transmural incorporation of the fish bone into the gastric wall.

**Fig. 5:**
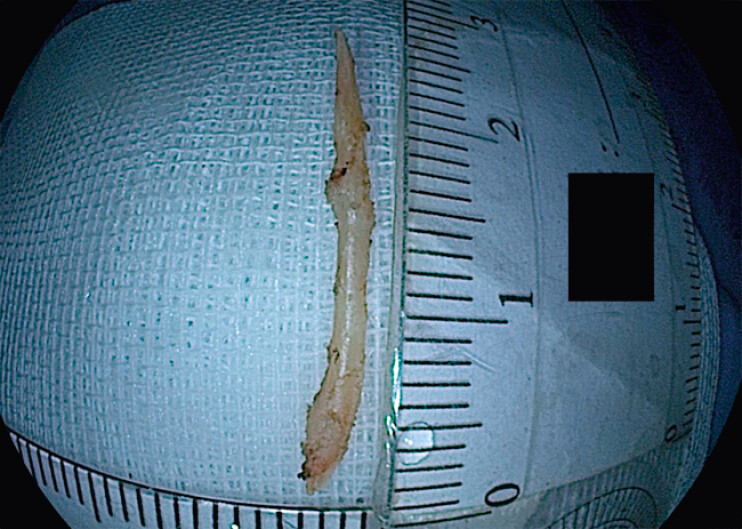
Retrieved fish bone, measuring approximately 3 cm in length.

A 38-year-old man underwent abdominal computed tomography (CT) during routine health screening, which revealed a linear hyperdense structure near the lesser curvature of the gastric antrum ( Fig. 1 ). He recalled ingesting a fish bone 2 months earlier but experienced no significant discomfort at the time and did not seek medical attention. Initial upper endoscopy showed no mucosal abnormalities ( Fig. 2 ). Endoscopic ultrasound (EUS) localized the foreign body within the deep muscularis propria ( Fig. 3 ). EUS-guided methylene blue injection and titanium clip placement were performed to guide endoscopic submucosal dissection, exposing the muscularis layer. Despite adequate dissection, the fish bone remained invisible, likely due to chronic inflammation and fibrosis resulting in transmural incorporation and concealment ( Fig. 4 ). Real-time EUS was re-employed for precise re-localization, followed by a deeper incision. The foreign body was tightly adherent to the gastric wall and could not be removed with forceps. A snare was used to anchor and provide countertraction, while a mucosal incision knife facilitated meticulous dissection of the fibrotic base. This coordinated traction–dissection approach enabled successful full-thickness endoscopic resection, retrieving a 3-cm fish bone ( Fig. 5 ). The defect was closed with titanium clips ( Video 1 ). This case highlights that fish bone ingestion warrants careful evaluation even where endoscopy findings are normal; additional imaging such as CT and the dynamic benefits of EUS play crucial roles.

Successful endoscopic full-thickness resection, guided by endoscopic ultrasound, of a fish bone undetected by endoscopy.Video 1

Endoscopy_UCTN_Code_TTT_1AO_2AL

